# Allelopathic effect of *Artemisia argyi* on the germination and growth of various weeds

**DOI:** 10.1038/s41598-021-83752-6

**Published:** 2021-02-22

**Authors:** Jinxin Li, Le Chen, Qiaohuan Chen, Yuhuan Miao, Zheng Peng, Bisheng Huang, Lanping Guo, Dahui Liu, Hongzhi Du

**Affiliations:** 1 Hubei Provincial Key Laboratory of Traditional Chinese Medicine Resources and Traditional Chinese Medicine Chemistry, Hubei University of Chinese Medicine, Wuhan, China; 2 National Resource Center for Chinese Materia Medica, China Academy of Chinese Medical Sciences, Beijing, China

**Keywords:** Ecology, Plant sciences

## Abstract

Allelopathy means that one plant produces chemical substances to affect the growth and development of other plants. Usually, allelochemicals can stimulate or inhibit the germination and growth of plants, which have been considered as potential strategy for drug development of environmentally friendly biological herbicides. Obviously, the discovery of plant materials with extensive sources, low cost and markedly allelopathic effect will have far-reaching ecological impacts as the biological herbicide. At present, a large number of researches have already reported that certain plant-derived allelochemicals can inhibit weed growth. In this study, the allelopathic effect of *Artemisia argyi* was investigated via a series of laboratory experiments and field trial. Firstly, water-soluble extracts exhibited the strongest allelopathic inhibitory effects on various plants under incubator conditions, after the different extracts authenticated by UPLC-Q-TOF-MS. Then, the allelopathic effect of the *A. argyi* was systematacially evaluated on the seed germination and growth of *Brassica pekinensis*, *Lactuca sativa*, *Oryza sativa*, *Portulaca oleracea*, *Oxalis corniculata* and *Setaria viridis* in pot experiments, it suggested that the *A. argyi* could inhibit both dicotyledons and monocotyledons not only by seed germination but also by seedling growth. Furthermore, field trial showed that the *A. argyi* significantly inhibited the growth of weeds in *Chrysanthemum morifolium* field with no adverse effect on the growth of *C. morifolium*. At last, RNA-Seq analysis and key gene detection analysis indicated that *A.argyi* inhibited the germination and growth of weed via multi-targets and multi-paths while the inhibiting of chlorophyll synthesis of target plants was one of the key mechanisms. In summary, the *A. argyi* was confirmed as a potential raw material for the development of preventive herbicides against various weeds in this research. Importantly, this discovery maybe provide scientific evidence for the research and development of environmentally friendly herbicides in the future.

## Introduction

The application of chemical pesticides has made substantial contributions to global agricultural development and saved countless people from hunger. However, in recent years, the overuse of chemical pesticides has resulted in excessive pesticide residues in agricultural products, causing serious environmental problems and posing a huge threat to human health^1^. Therefore, the concepts of “ecological farming” and “organic products” were proposed to reduce the use of chemical fertilizers and pesticides and to develop new, safe, effective and pollution-free substitutes. Thus, quite a few green pesticides and herbicides have been applied to meet the societal needs.

Allelopathy, also called the interaction effect, means that chemical substances produced by certain plants then affect the growing development of other plants^2^. Allelochemicals can stimulate or inhibit the germination or/and growth of plants, and increase the resistance of crops to biotic and abiotic stress. Proverbially, plant-derived allelochemicals do not exert residual or toxic effects. Therefore, they are considered as the perfect substitutes for synthetic herbicides^3^. As early as the late nineteenth century, researchers occasionally found that weeds were not grew near the walnut trees. Based on this interesting phenomenon, scientists revealed that the walnut tree secretes walnut quinone to inhibit the growth of surrounding weeds. In the 1980s, sorghum quinones released from sorghum roots were also reported to effectively restrain the germination and growth of *Abutilon theophrasti*, *Amaranthus retroflexus*, *Echinochloa crusgalli*, *Digitaria sanguinalis* and *Setaria viridis*. Obviously, studies of plant-derived herbicides based on allelopathy were gradually reported.

To date, numerous commercial herbicides have been successfully discovered from plants or plant extracts worldwide. In 1996, Cook isolated a compound called strigol from cotton root secretions which effectively promoted the germination of *Striga asiatica* seeds, the strigol treatment induced the “suicidal germination” of *Striga asiatica*, *Orobanche coerulescens* and other parasitic weeds in the absence of hosts^4^. Moreover, the trigoxazonane secreted by *Trigonella foenum-graecum* root system can effectively inhibit the seeds germination of *Orobanche crenata* (a malignant weed in the field of leguminous crops)^5^. In addition, a *Medicago sativa* extract also showed a certain concentration-dependent inhibiting effect on the germination and growth of *Elymus nutans* seeds^6^. In brief, plant-derived allelochemicals have high research value and broad application prospects. Therefore, more plant materials with a wide range of sources, low cost and markedly allelopathic effects are urgently explored to be bioherbicide.

*Artemisia argyi*, a perennial herbaceous plant widely known in Asia, is a renowned traditional medicinal material used in China as the treatment of pain, bleeding and eczema^7^. *A. argyi* is rich in secondary metabolites, including many chemical constituents which are beneficial to human health, such as volatile oils, flavonoids, tannins, polysaccharides and phenolic acid. From ancient times, *A. argyi* is mainly used to produce moxa stick for moxibustion to prevent and treat multifarious disease in China, Japan and Korea. Moxa are obtained by crushing, sifting, mashing and sieving *A. argyi* leaves. Typically, 5 to 30 kg of *A. argyi* leaves can produce only 1 kg of moxa. The remaining wastes comprise the mesophyll tissue, petiole, vein and other fine debris of *A. argyi* leaves are commonly called as *A. argyi* leaf powder^8^. According to preliminary statistics, about 11,160,000 kg of *A. argyi* powder were produced every year in China, needing to be dealt with urgently. However, *A. argyi* leaf powder has not yet been widely consumed in industry. Meanwhile, the storage of *A. argyi* powder occupies a large amount of factory space and the disposal of *A. argyi* powder is costly to the industry. Thus, comprehensive utilization of *A. argyi* powder is urgently to be expanded for the industry of *A. argyi* to conduct waste disposal.

In a previous study, we plan to exploit the *A. argyi* leaf powder as fertilizer in a *Chrysanthemum morifolium* field. Coincidently, we found that *A. argyi* powder could significantly inhibit the germination of weeds and reduce the varieties and biomass of weeds in experimental field. In further investigation in local area, we are also told that the waste water of *A argyi* essentia oil industry resulted in the inhibition of germination of plant seeds when the effluent was discharged into the farmland directly. Therefore, we speculated that certain allelochemicals present in *A. argyi* might inhibit weed growth. In the present study, different solvents, including distilled deionized water, 50% ethanol and pure ethanol, were used to extract allelochemicals in *A. argyi* and the ingredients were authenticated by UPLC-Q-TOF-MS. Meanwhile, the results showed that the water-soluble extracts exhibit the strongest allelopathic inhibitory effect on the germination of *Brassica pekinensis*, *Lactuca sativa* and *Oryza sativa* seeds. Pot experiments suggested that both dicotyledons and monocotyledons could be inhibited by seed germination and growth. Furthermore, field experiments also confirmed that *A. argyi* exhibited a significant inhibitory effect on the germination and growth of weed seeds. At last, transcriptomics analysis indicated that *A.argyi* inhibited the germination and growth of weed via multi-targets and multi-paths while the inhibiting of the chlorophyll synthesis of target plants was confirmed as one of the key mechanisms. In summary, this study not only reveals the inhibitory effect of *A. argyi* on weeds but also may provide the scientific evidence for the development of an environmentally friendly herbicide.

## Results

### The chemical components analysis of different extracts of *A. argyi*

In our preliminary study, we accidentally found that *A. argyi* powder significantly inhibited the germination and reduced the varieties and biomass of weeds in the field, when it was applied as a fertilizer originally. Therefore, we speculated that certain allelochemicals present in *A. argyi* might inhibit the growth of weeds. To investigated the possible allelochemicals in *A. argyi*, three solvents (water, 50% ethanol and pure ethanol) were used to extract the metabolites in *A. argyi* leaves. The three type of extracts were analysed by UPLC-Q-TOF-MS and the components were confirmed by comparison with synthetic standards and MS data in literatures^9–11^. As shown in Table 1 and supplement Fig. 1, we have identified a total of 29 components in *A. argyi*. Six main compound mass signals were identified in the water extract: caffeic acid, schaftoside, 4-caffeoylquinic acid, 5-caffeoylquinic acid, 3,5-dicaffeoylquinic acid and 3-caffeoylquinic acid. The main compounds of the 50% ethanol extract were 4,5-dicaffeoylquinic acid, 3-caffeoylquinic acid, schaftoside, rutin, kaempferol 3-rutinoside, 3,4-dicaffeoylquinic acid, 3,5-dicaffeoylquinic acid, 3-caffeoy,1-p-coumaroylquinic acid, 1,3,4-tri-caffeoylquinic acid and eupatilin. The metabolites with higher contents in the pure ethanol extract were eupatilin, jaceosidin and casticin. Among these compounds, caffeic acid is very unique in water extract. Higher contents of schaftoside, 4-caffeoylquinic acid and 3-caffeoylquinic acid were observed in water extract and 50% ethanol extract, but very low concentrations were detected in the pure ethanol extract. 3,4-dicaffeoylquinic acid, jaceosidin, eupatilin and casticin were present at higher concentrations in the 50% ethanol extract and pure ethanol extract, but were detected at very low concentrations or were absent in the water-soluble extract. In a word, we have preliminarily identified the chemical components of different extracts.

**Table 1:** The chemical composition of different solvent extracts of *A. argyi.*

Compd	Rt/min	Molecular Formula	Exptl.[M − H]^−^	MS/MS fragmentation	Identification	Water-soluble extract	50% ethanol extract	Pure ethanol extract
1	3.26	C_16_H_18_O_9_	353.0842	191.0515, 179.0294, 173.0415, 161.0181, 135.0404	5-Caffeoylquinic acid*	+	+	–
2	4.40	C_16_H_18_O_9_	353.0839	191.0511, 179.0305, 173.0391, 161.0198, 135.0403	3-Caffeoylquinic acid*	+	+ +	–
3	4.64	C_16_H_18_O_9_	353.0845	191.0513, 179.0302, 173.0407, 161.0191, 135.0400	4-Caffeoylquinic acid*	+	+	–
4	4.87	C_9_H_8_O_4_	179.0304	135.0402, 134.0323, 89.0333	Caffeic acid*	+ + +	–	–
5	4.99	C_12_H_18_O_7_S	305.0665	225.1123, 96.9574	Hydroxyjasmonic acid-O-sulphate	+	+	–
6	5.37	C_18_H_28_O_9_	387.1636	207.0992	Hydroxyjasmonic acid hexose	+	+	–
7	5.75	C_27_H_30_O_15_	593.1507	533.1297, 503.1192, 473.1214, 383.0719, 353.0681, 117.0279	Apigenin-6,8-di-C-glucoside	+	+	–
8	6.07	C_17_H_20_O_9_	367.1016	193.0591, 191.0462, 173.0442	5-Feruoyl quinic acid	+	+	+
9	6.52	C_26_H_28_O_14_	563.1412	473.1059, 443.0958, 383.0724, 353.0629	Schaftoside*	+ +	+ +	–
10	6.66	C_15_H_18_O_5_	277.1041	259.0922, 233.1140, 215.1024	Artemargyinolide C	+	+	–
11	6.97	C_26_H_28_O_14_	563.1412	473.1059, 433.0950, 383.0627, 353.0685	Isoschaftoside*	–	–	–
12	7.39	C_27_H_30_O_16_	609.1473	301.0245, 300.0179	Rutin	–	+ +	+
13	7.52	C_27_H_30_O_15_	593.1508	285.0309, 284.0468, 133.0291	Kaempferol 3-rutinoside	+	+ +	–
14	7.65	C_21_H_20_O_12_	463.0862	301.0215, 300.0212, 271.0225, 255.0222	Quercetin − 3-O-glucoside	–	+	–
15	8.23	C_25_H_24_O_12_	515.1187	353.0829, 335.0738, 191.0505, 179.0306, 173.0407, 161.0189, 135.0399	3,4-Dicaffeoylquinic acid*	–	+ +	+
16	8.45	C_25_H_24_O_12_	515.1193	353.0845, 335.0781, 191.0516, 179.0315, 173.0442, 161.0166	1,5-Dicaffeoylquinic acid	+	+	+
17	8.57	C_25_H_24_O_12_	515.1188	353.0844, 335.0748, 191.0512, 179.0299, 173.0408, 161.0189, 135.0394	3,5-Dicaffeoylquinic acid*	+	+ +	+
18	9.18	C_21_H_18_O_11_	445.0754	270.0433, 269.0479	Apigenin-O-glycuronide	+	+	-
19	9.52	C_25_H_24_O_12_	515.1179	353.0842, 335.0720, 191.0512, 179.0302, 173.0408, 161.0196, 135.0394	4,5-Dicaffeoylquinic acid*	+	+ + +	+
20	10.27	C_25_H_24_O_11_	499.1234	353.0838, 337.0976,319.0792,191.0520	3-Caffeoy,1-p-coumaroylquinic acid	–	+ +	–
21	11.28	C_15_H_12_O_6_	287.0518	151.0020, 135.0375	Dihydrokaempferol	–	+	+
22	11.88	C_15_H_10_O_6_	285.0367	175.O373, 151.0083, 133.0291,107.0193	Luteolin	–	+	–
23	12.61	C_34_H_30_O_15_	677.1521	515.1199, 497.1191, 353.0866, 335.0796, 191.0554, 179.0348, 173.0350, 161.0194	1,3,4-Tri-caffeoylquinic acid	–	+ +	–
24	14.07	C_15_H_10_O_5_	269.0419	151.0050, 117.0307	Apigenin*	+	+	+
25	14.46	C_16_H_12_O_6_	299.0526	285.0350, 284.0288, 256.0336, 227.0304, 212.0421, 186.0281, 136.9832	Hispidulin	–	+	+
26	15.11	C_17_H_14_O_7_	329.0632	314.0396, 299.0154, 145.0239	Jaceosidin*	–	+	+ +
27	17.36	C_17_H_14_O_6_	313.0676	298.0424, 283.0257	Dihydroxy-dimethoxy flavone	–	+	+
28	17.88	C_18_H_16_O_7_	343.0848	328.0480, 313.0371, 298.0146, 132.0202	Eupatilin*	+	+ +	+ + +
29	18.73	C_19_H_18_O_8_	373.0907	358.0674, 343.0438, 328.0193, 315.0466, 285.0003, 257.0053, 229.0100, 201.0140, 173.0180, 145.0226, 117.0275	Casticin*	–	+	+

“ +  +  + , +  + , + ” means that the relative content is from high to low, “–”means very low content; Compd. was the compound number; Rt represtented retention time; Exptl. [M-H]- was experimental m/z of molecular ions in the negative ionisation mode; *Compound was positively identified by comparing the retention time, high-resolution molecular ions and fragment ions with the authentic standards. The mass spectrometry data such as total ion chromatograms, molecular ion peaks and secondary fragment ions were collected by Masslynx 4.1 software and processed with ProGenesis QI V2.0 (Waters Corp., Milford, USA).

### Comparison of the allelopathic effects of different extracts of *A. argyi*

**Figure 1:**
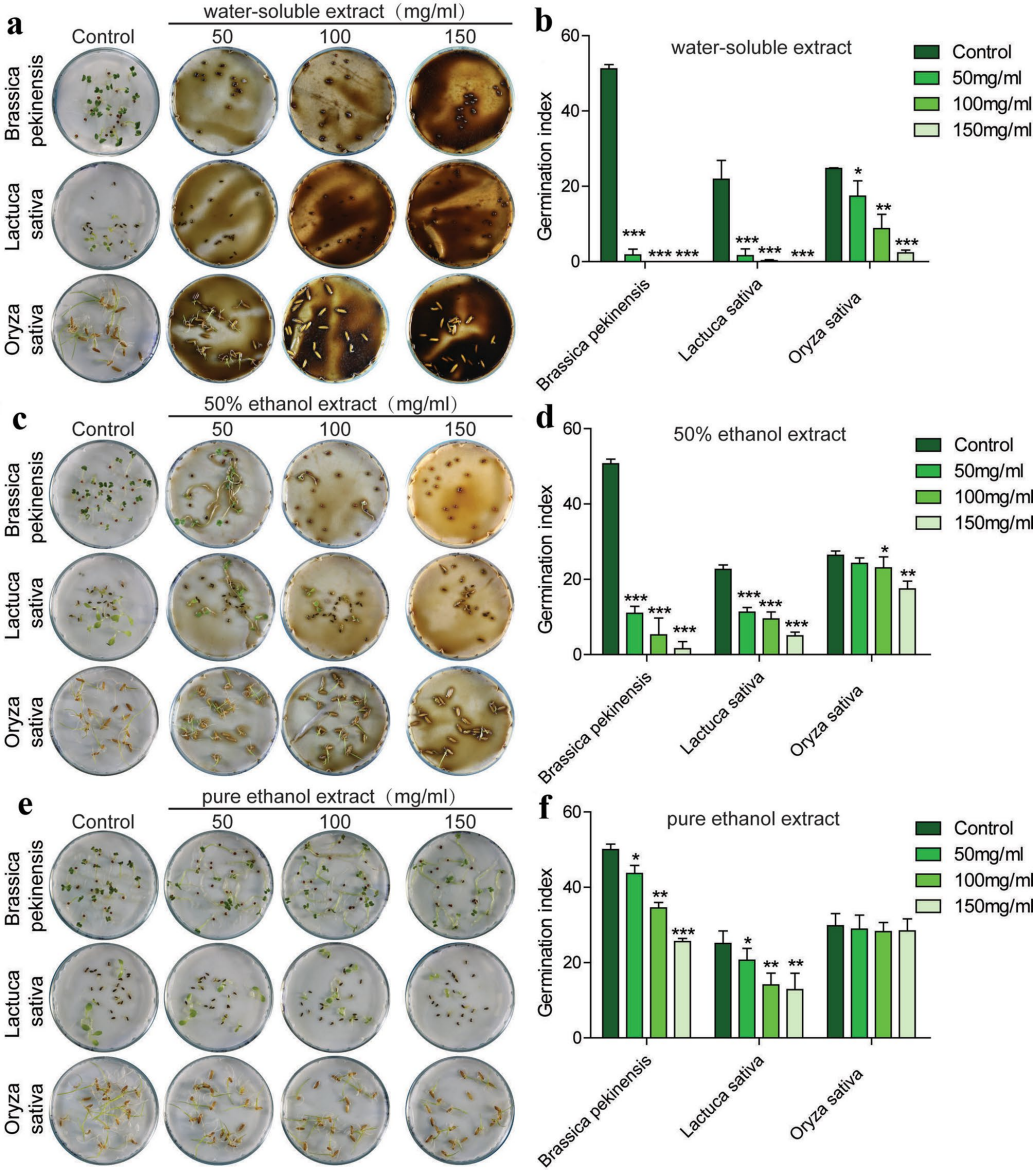
The different solvent exracts of *A. argyi*: (**a**,**b**) the water-soluble extract, (**c**,**d**) the 50% ethanol extract,and (**e**,**f**) the pure ethanol extract exert allelopathic effects on germination index of different plants. (n = 3,*P < 0.05, **P < 0.01, ***P < 0.001).

**Figure 2:**
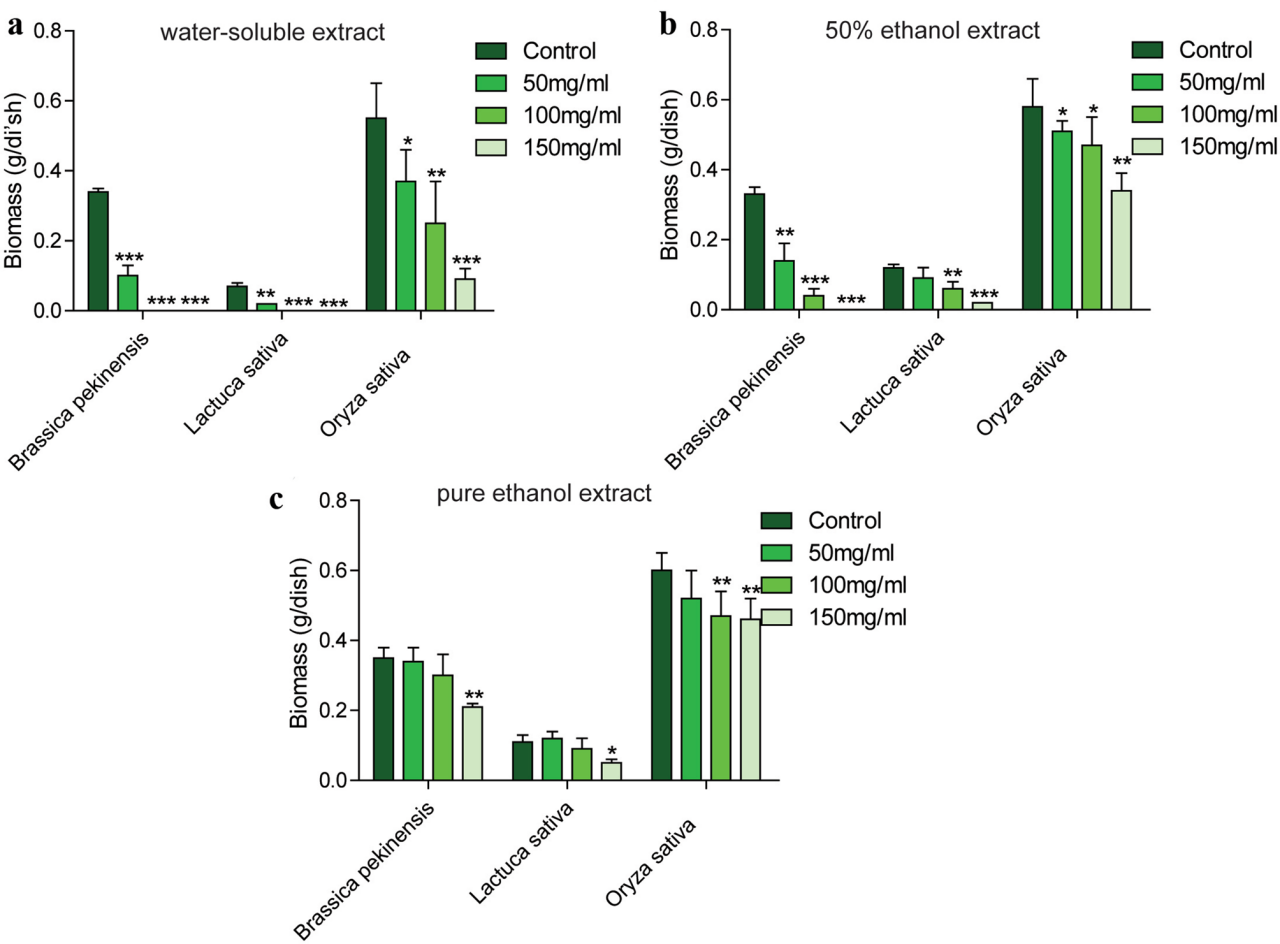
Allelopathic effects of the water-soluble extract (**a**), the 50% ethanol extract (**b**),and the pure ethanol extract(c) from *A. argyi* with different concentrations on biomass of three plants were compared. (n = 3,*P < 0.05, **P < 0.01, ***P < 0.001).

To explore the allelopathic effects of three different extracts of *A. argyi*, seed germination and seedling growth of *B. pekinensis*, *L. sativa* and *O. sativa* were investigated after treatment of *A. argyi* powder extracts. The results showed that the allelopathic inhibition increased in a concentration dependent manner. When seeds were incubated with extracts in a range of concentrations, the water-soluble extract of *A. argyi* powder exerted an extremely significant inhibitory effect on the germination index of all the three plants (Fig. 1a,b). While the 50% ethanol extract also showed striking allelopathic inhibitory effects on the germination index of *B. pekinensis* and *L. sativa*, but moderately inhibitory effects on *O. sativa* (Fig. 1c,d). Similarly, the pure ethanol extract only showed powerful inhibitory effects on the germination index of *B. pekinensis* and *L. sativa*, but no effects on *O. sativa* (Fig. 1e,f). Additionally, the water-soluble extract of *A. argyi* powder displayed extremely inhibition of the biomass of the three plants (Fig. 2a), while the 50% ethanol extract also exerted extremely significant allelopathic inhibitory effects on the biomass of *B. pekinensis* and *L. sativa* but inhibited *O. sativa* moderately (Fig. 2b). However, the pure ethanol extract exerted inhibitory effects on the biomass of these three plants only in high concentrations (Fig. 2c). Based on these results, the allelopathic intensity of the three different extracts of *A. argyi* was in the order of water-soluble extract > 50% ethanol extract > pure ethanol extract.

### The water-soluble extract of *A. argyi* inhibited the germination and growth of different plants

**Figure 3:**
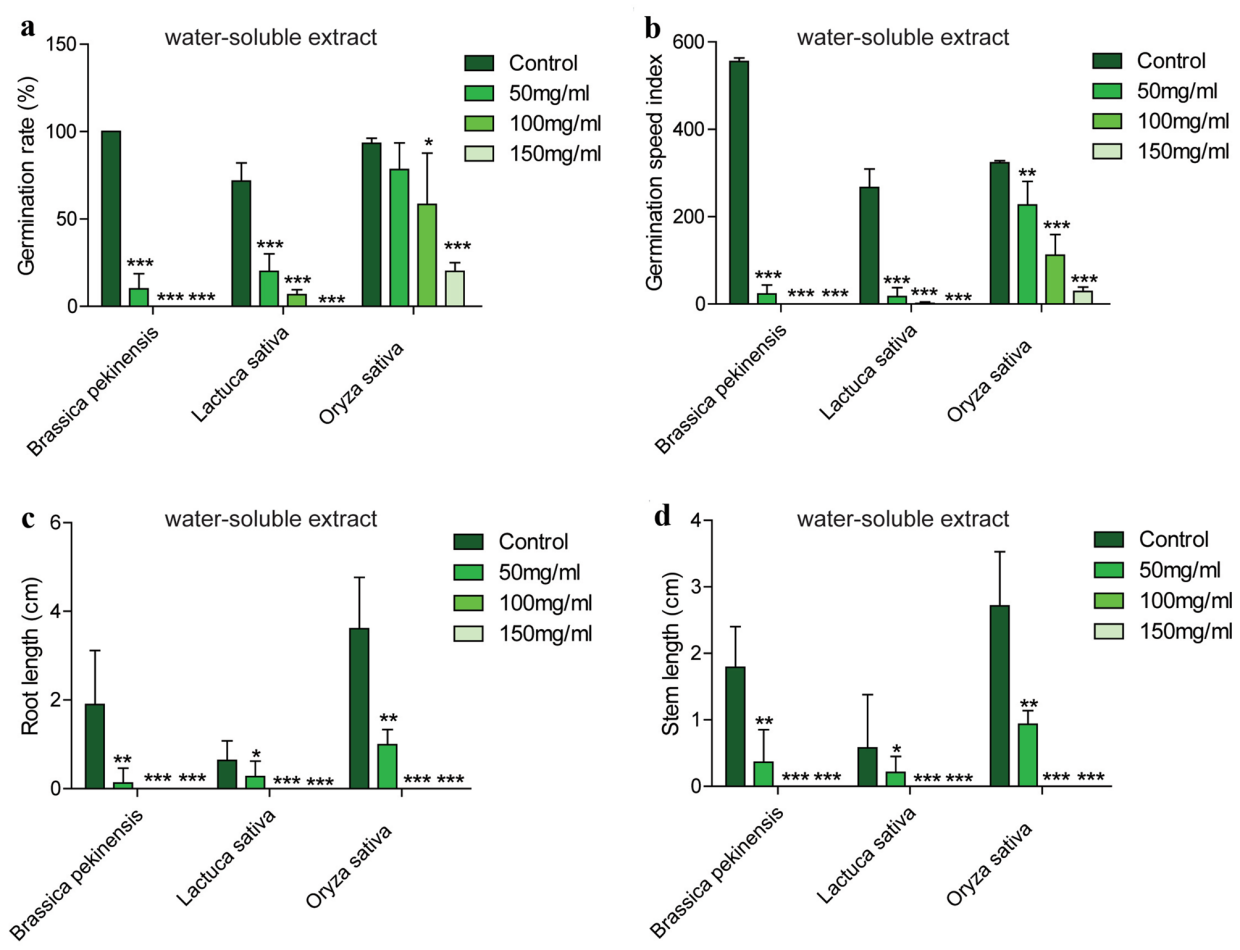
The water-soluble extract of *A. argyi* inhibits the germination and growth of *Brassica pekinensis*, *Lactuca sativa* and *Oryza sativa*. Specific performance is in a series of indicators: (**a**) the germination rate, (**b**) the germination speed index, (**c**) the root length, (**d**) the stem length. (n = 3,*P < 0.05, **P < 0.01, ***P < 0.001).

From the above results, water-soluble extract of *A. argyi* powder exhibited the strongest allelopathic effects on plant growth. To evaluate systematacially, the germination rate, germination rate index, germination index, root length, stem length and biomass were selected as the allelopathic response indexes as previously reported^12,13^. As shown in Fig. 3 and Table 2, the germination rate, germination speed index, germination index, root length, stem length and biomass of *B. pekinensis* could be significantly inhibited by a low concentration extract (50 mg/ml), and the order of inhibition efficiency was: germination index > germination speed index > root length > germination rate > stem length > biomass. When the treatment concentration up to 100 mg/ml, all the allelopathic indexes were -1.00 which indicated that no seeds could germinate under this treatment concentration. For *L. sativa,* the order of inhibition efficiency was germination speed index > germination index > biomass > germination rate > root length > stem length. All allelopathy response indexes reached -1.00 when plants were treated with 150 mg/ml extract. For *O. sativa,* the six physiological indexes also could be inhibited by a low concentration of extract (50 mg/ml), but the changes were not as obvious as the changes in *B. pekinensis* and *L. sativa*. The intensity of inhibition on the six indexes was root length > stem length > biomass > germination index > germination speed index > germination rate. When *O. sativa* seeds treated with 100 mg/ml of extract, the allelopathic response index of root length and stem length were -1.00. The germination rate, germination speed index, germination index and biomass were -0.79, -0.91, -0.91 and -0.84, respectively, under the treatment with 150 mg/ml of extract. In brief, according to the comprehensive allelopathy index of the 6 indicators , the order in which they were sensitive to water-soluble extract of *A. argyi* were *B. pekinensis* (Cruciferae) > *L. sativa* (Compositae) > *O. sativa* (Gramineae).

**Table 2:**
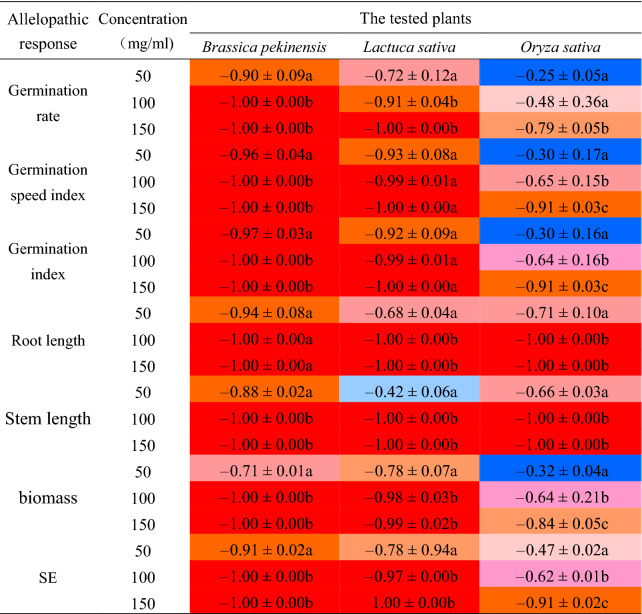
Allelopathic response indexes of the water-cooled extract of *A. argyi* on different tested plants.

(The color is near to deep red, it means that the Allelopathic response indexes is closer to - 1; the color is near to deep blue, it means that the Allelopathic response indexes is closer to 0. Different letters in the same column showed significant differences, with a significant level of 5%).

### *A.argyi* inhibited the germination and growth of different plants in pot experiment

**Figure 4:**
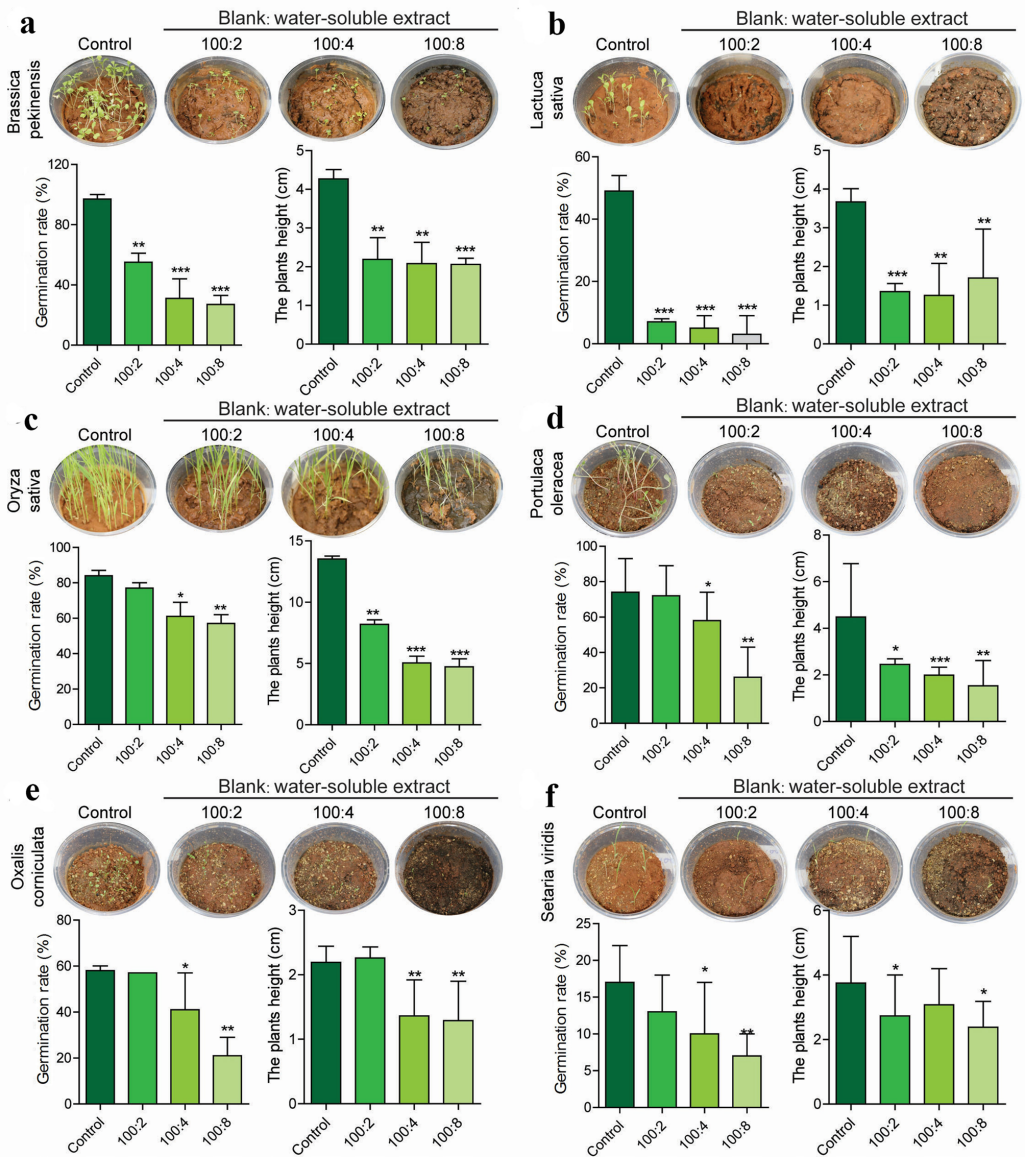
Mean germination index and plants height plus standard deviation for the six species with different proportions of *A. argyi* treatment in pot experiment: *Brassica pekinensis* (**a**), *Lactuca sativa* (**b**), *Oryza sativa* (**c**), *Portulaca oleracea* (**d**), *Oxalis corniculata* (**e**), *Setaria viridis* (**f**). (n = 3,*P < 0.05, **P < 0.01, ***P < 0.001).

Further, the allelopathic effects of *A. argyi* were evaluated in pot experiments via soil mixed with a certain proportion of *A. argyi* powder. Firstly, seeds of *B. pekinensis*, *L. sativa*, *O. sativa*, *P. oleracea* , *O. corniculata* and *S. viridis* were sown into the mixed soil to observe the effects of *A. argyi* on seeds germination and plant growth. As shown in Fig. 4, when the proportion of soil: *A. argyi* powder was 100:2, the germination rate of *B. pekinensis* and *L. sativa* was significantly inhibited, and the plant height of *B. pekinensis*, *L. sativa, O. sativa* and *P. oleracea* was also inhibited. As the proportion of *A. argyi* powder gradually enhanced, the level of inhibition of the germination rate and plant height of these six tested plants was gradually increased. When the ratio reached 100:8, the germination inhibition rates of *B. pekinensis*, *L. sativa*, *O. sativa, P. oleracea*, *O. corniculata* and *S. viridis* were 71.82%, 93.20%, 31.75%, 65.47%, 63.60% and 60.78%, respectively. The plant height inhibition rates were 51.76%, 71.39%, 64.99%, 65.70%, 40.94%, and 36.53%, respectively, and the leaves turn yellow gradually in many plants. Therefore, the results from the laboratory were remarkable, it is urgent to verify the *A.argyi* powder as a weed herbicide in the field.

### *A.argyi* inhibited the germination and growth of weeds in *Chrysanthemum morifolium* field

**Figure 5:**
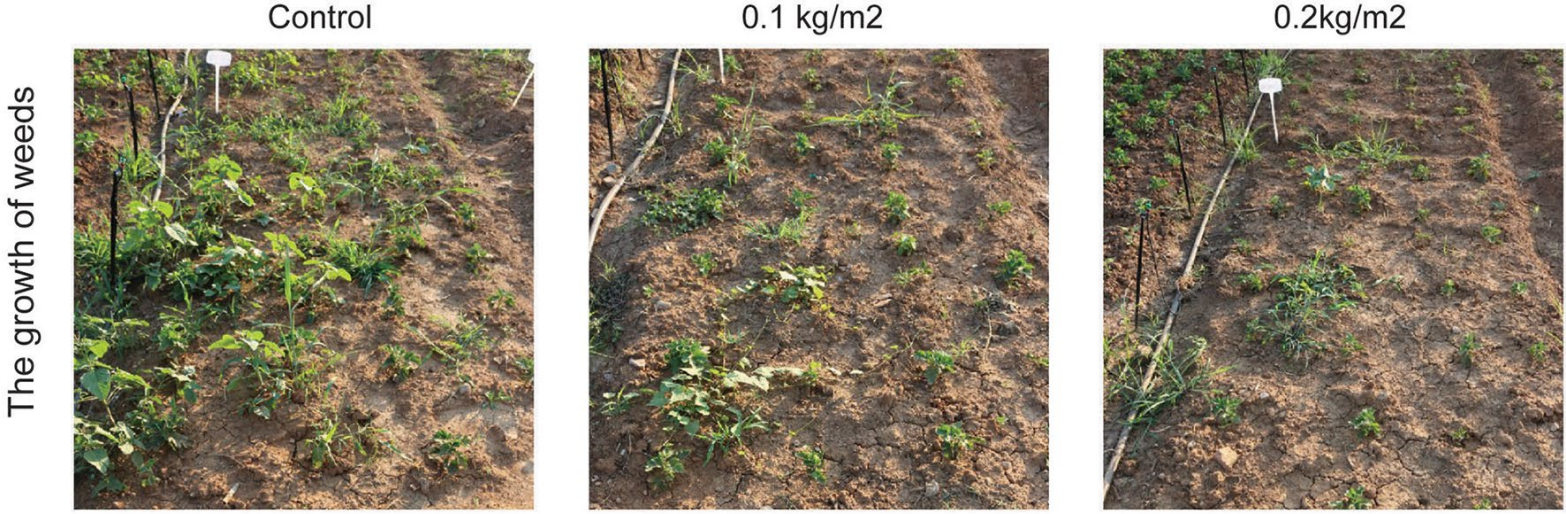
Actual growth of weeds in *Chrysanthemum morifolium* field with *A. argyi* treatment.

Then, *A. argyi* powder was applied into the *C. morifolium* field to explore the inhibition of weeds and evaluate the adverse effect on crops. After the application of *A. argyi* powder for one month, six species of weeds appeared in the control group, while 3 species of weeds appeared in the group treated with 0.1 kg/m^2^ powder, and only 1 species of weeds appeared in the group treated with 0.2 kg/m^2^ powder (Fig. 5). Our previous report^14^ showed that the inhibition rate of weeds species were 50% and 83.33%, respectively, in the low dose group and the high dose group. Furthermore, the quantity and biomass of weeds were inhibited by 46.61% and 60.98% in the 0.1 kg/m^2^ treatment group compared with control group^14^. The weed quantity and biomass were inhibited by 60.90% and 82.11%, respectively, in the 0.2 kg/m^2^ treatment group^14^. In addition, *C. morifolium* grew very well in the field with no growth inhibition by *A. argyi* powder. After *C. morifolium* was harvested in the autumn, there were no significant differences in the number of flowers and the weight of flowers between the experimental group and the blank control group. Therefore, *A. argyi* powder did not inhibit the growth of the existing crops in the field, but only exerted an obvious effect on the ungerminated weed seeds in the field. Importantly, *A. argyi* powder may be a potential raw material for developing safe and environmentally friendly plant-sourced herbicides.

### *A.argyi* inhibited the germination and growth of weed via the suppression of chlorophyll synthesis and photosynthesis

**Figure 6:**
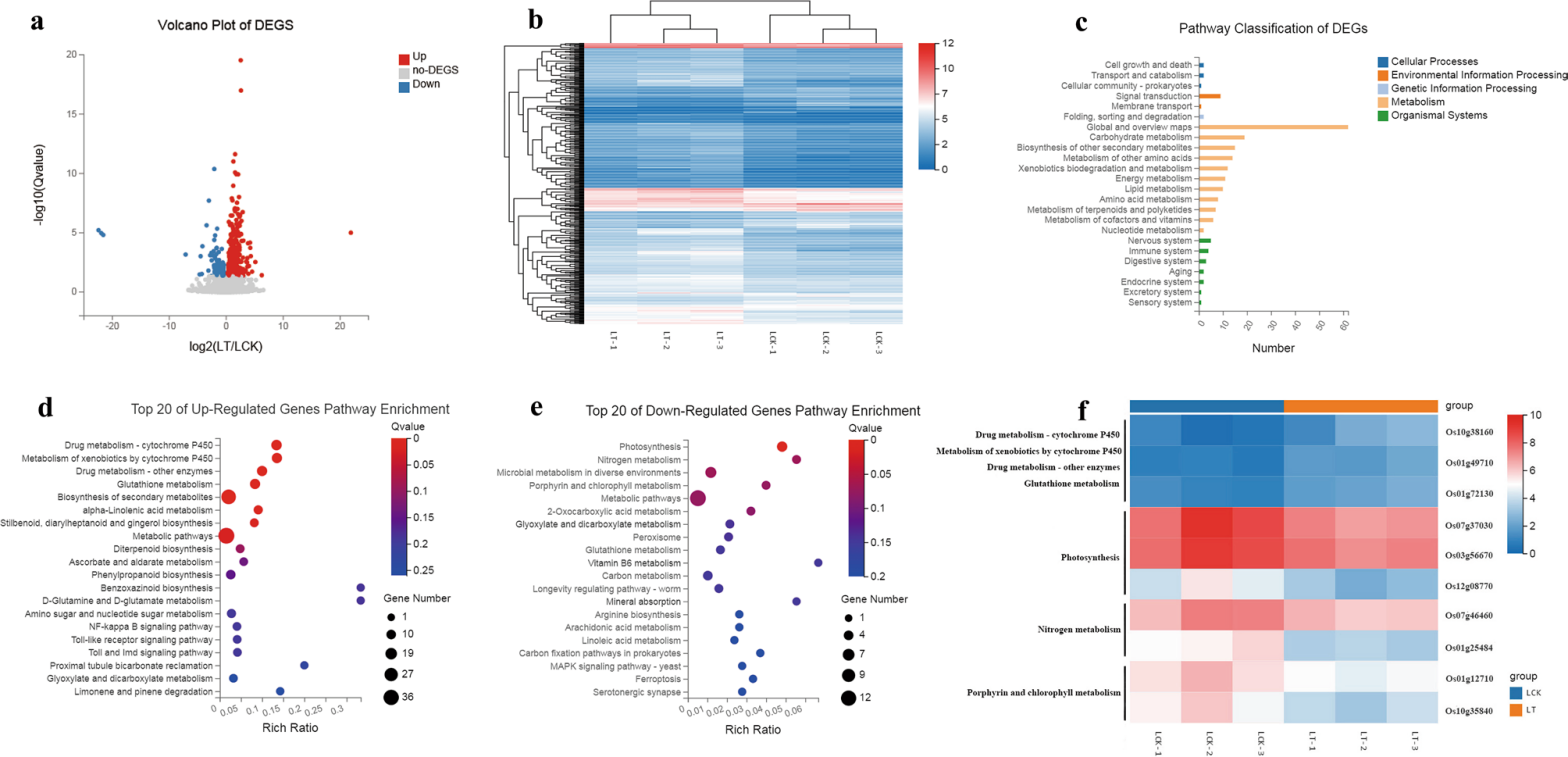
RNA-Seq Analysis of the *O. sativa* under two treatment models. (**a**) The volcano plot of DEGs. Different colors represent different gene expression trends. (**b**) Hierarchical cluster analysis of the significantly changed DEGs. The color key represents FPKM-normalized log2-transformed counts. (**c**) KEGG pathway classification analysis of DEGs. (**d**) Top 20 KEGG pathway enrichment analysis of up-regulated DEGs. The colors are shaded according to the Q-values level, as shown in the color bars gradually from low(red) to high(blue); the size of the circle indicates the number of DEGs from small (less) to big (more); the same of next figure. (**e**)Top 20 KEGG pathway enrichment analysis of down-regulated DEGs. (**f**) The expression of key DEGs in key pathways. The color key represents FPKM-normalized log2-transformed counts.

The inhibitory effect of weeds has been well documented, but the mechanism is urgently to be revealed. As reported^15^, transcriptomic analysis is the rapid and objective method to explore the mechanism. For the convenience of transcriptome analysis, we choosed the accepted model plant *Oryza sativa* as the research object, based on the BGISEQ500 platform. Each sample produced an average of 6.76G of data, with a total of 87.42% of the reads were mapped to the reference genome. Further, genome-wide gene expression profiles were compared between *A.argyi*- and water-treated wild-type plants. In total, 311 differentially expressed genes (DEGs) with (log2|FC (ratio of treated/control)|≥ 1, *p*-value < 0.05) were selected for further investigation. Among them, 245 genes were up-regulated and 66 genes were down-regulated in the *A.argyi*-treated plants (Fig. 6a,b).

To elucidate the potential biological functions of the DEGs, Kyoto Encyclopedia of Genes and Genomes (KEGG) databases were used for pathway classification and enrichment analysis. The results showed that a maximum number of genes were classified in the metabolism category, followed by organismal systems, environmental information processing, cellular processes and genetic information processing (Fig. 6c). Furthermore, KEGG pathways of up- and down-regulated DEGs were investigated separately. For up-regulated DEGs, most genes were enriched in "Metabolic pathways" and “Biosynthesis of secondary metabolites”. In detail, "Drug metabolism—cytochrome P450"(11 unigenes), "Metabolism of xenobiotics by cytochrome P450" (10 unigenes), "Drug metabolism—other enzymes" (10 unigenes), and "Glutathione metabolism" (10 unigenes) , were the most significant enriched pathways(Fig. 6d). For down-regulated DEGs, Photosynthesis, Nitrogen metabolism and Porphyrin and chlorophyll metabolism pathways were the most significant enriched pathways (Fig. 6e). Specifically, 4 unigenes involved in Photosynthesis, 2 unigenes involved Nitrogen metabolism and 2 unigenes involved in Porphyrin and chlorophyll metabolism were down regulated upon *A.argyi* treatment (Fig. 6f). In summary, these results suggest that *A.argyi* inhibited the germination and growth of weed via multi-targets and multi-pathways, especially, inhibiting the photosynthesis and chlorophyll synthesis pathways.

**Figure 7:**
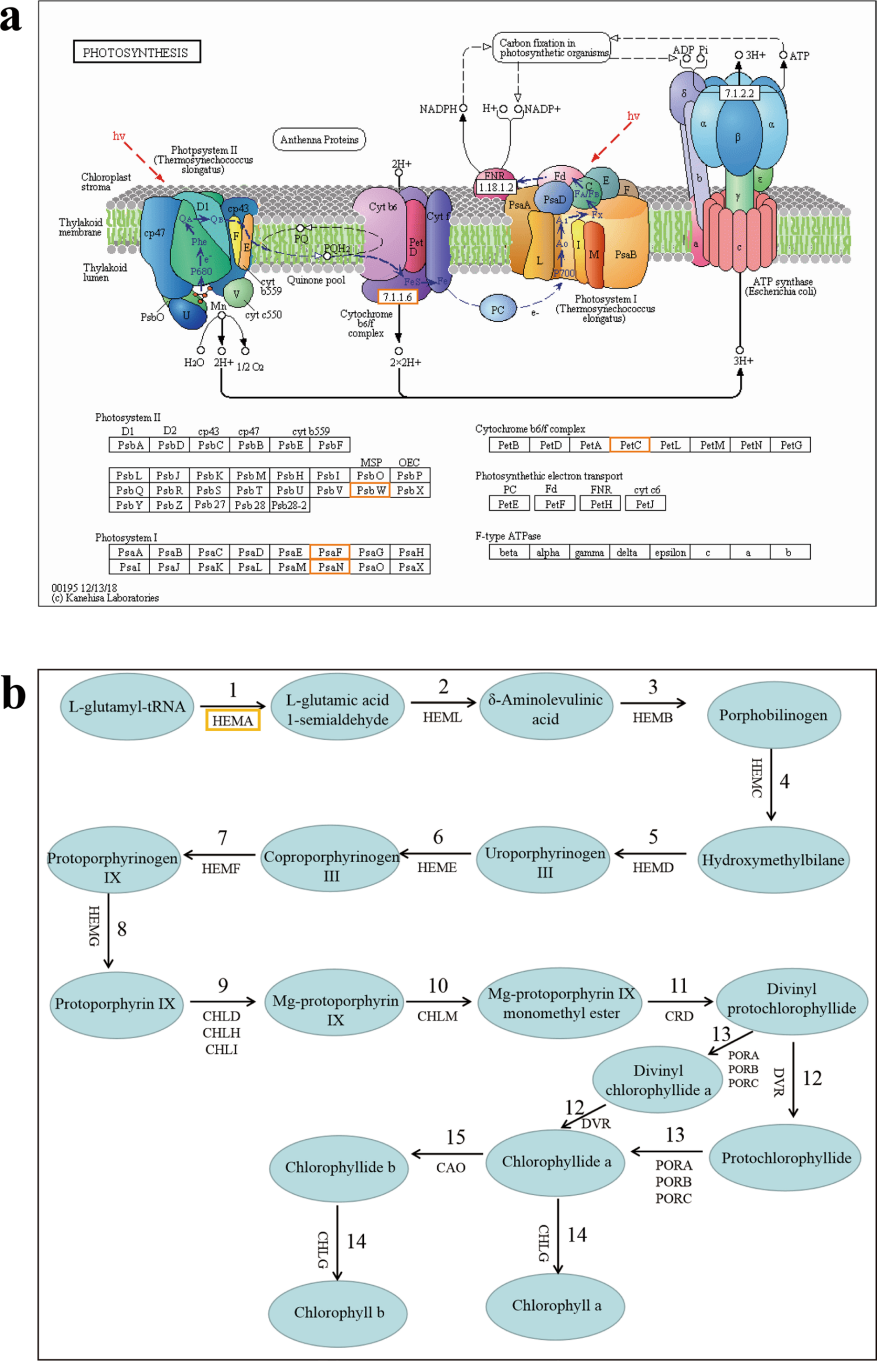
The down-regulated DEGs of “ photosynthesis” pathway (**a**) and “chlorophyll biosynthesis” pathway (**b**).

Photosynthesis is one of the most important physiological activities of plants. Our transcriptome analysis showed that photosynthesis might be the targets that inhibited by *A.argyi* treatment. Meanwhile, we also observed that *A.argyi* treatment caused the leaves of weeds turned yellow gradually in the pot experiment. The down-regulated genes of photosynthesis were involved in “photosystem II”, “photosystem I” and “cytochrome b6/f complex” (Fig. 7a)^16^. And the DEGs of porphyrin and chlorophyll metabolism were mainly involved in the preceding part of chlorophyll synthesis pathway (Fig. 7b)^17^. Therefore, we speculated that suppression of chlorophyll synthesis and photosynthesis was one of the key mechanism of *A.argyi* to inhibit weeds.

**Figure 8:**
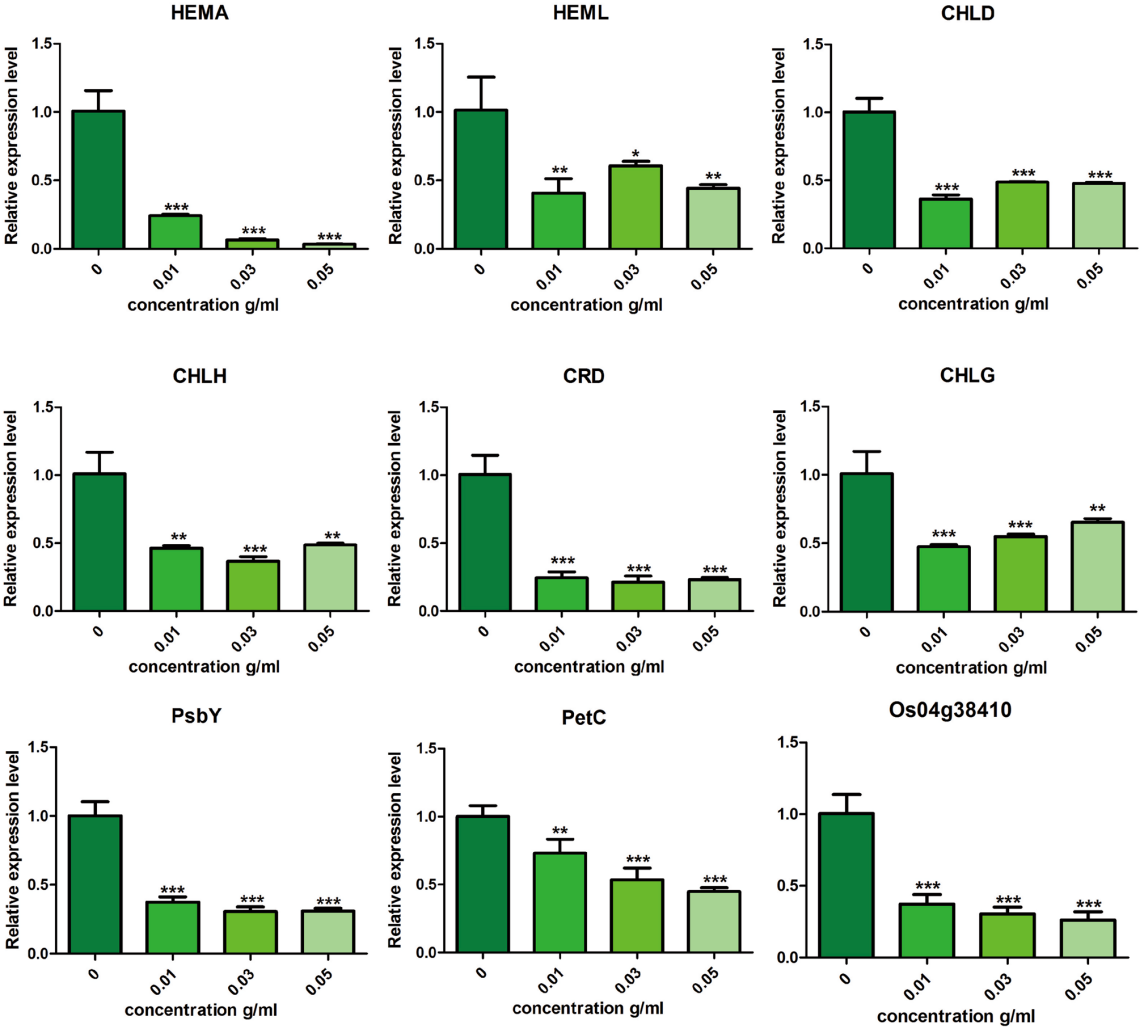
Expression of photosynthetic genes in *O. sativa* treated with different concentrations water-souble extact of *A.argyi.*

The key genes involved in photosynthesis pathways were verified by RT-qPCR, and the results were in consistent with our transcriptome analysis. As shown in Fig. 8, nine genes (HEMA “encoding glutamyl-tRNA reductase”, HEML “encoding glutamate-1-semialdehyde aminotransferase”, CHLD “encoding Mg chelatase D subunit”, CHLH “encoding Mg chelatase H subunit”, CRD “encoding Mg-protoporphyrinogen IX monomethylester cyclase”, CHLG “encoding chlorophyll synthase”, PsbY “encoding a polyprotein of photosystem II”, PetC “encoding the polypeptide binding the Rieske FeS center”, Os04g38410 “encoding subunits of the LHCII complex”) of photosynthesis were significantly suppressed by *A.argyi* treatment in a concentration dependent manner. Our transcriptome data and RT-qPCR verification showed that the suppression of chlorophyll synthesis and photosynthesis was one of the key mechanism of *A.argyi* ’s inhibitory effect on weeds.

## Discussion

### Water-soluble metabolites might be the allelochemicals of *A. argyi*

This study aimed to investigate whether the *A. argyi* can inhibit the germination and growth of other weeds. The Fig. 1 showed that the water-soluble extract exhibited the strongest inhibitory effect on seed germination and seedling growth of *B. pekinensis*, *L. sativa* and *O. sativa*, compared to the 50% ethanol and pure ethanol extract. As reported^18,19^, different extract of the same medicinal materials often obtains diverse components then results in different effects. As shown in Figs. 1 and 2, it suggested that the allelopathy of *A. argyi* might be mediated mainly by water-soluble compounds, indicating the possibility of practical application. According to abundant studies^20–22^, allelopathic substances are secondary metabolites of plants, most of which are synthesized via the shikimic acidand isoprene metabolic pathways. At present, known allelopathic substances mainly include phenols, quinones, coumarins, flavonoids, terpenes, sugars, glycosides, alkaloids and non-protein amino acids^23^. *A. argyi*, a perennial herbaceous plant is rich in secondary metabolites including volatile oils, flavonoids, polysaccharides, tannins, terpenes and trace elements^24^. Therefore, different solvent extracts of *A. argyi* were analyzed by UPLC-Q-TOF-MS as shown in Table 1 and supplement Fig. 1. As a typical phenolic acid, caffeic acid is several times more abundant than other allelopathic substances in the water extract. Published research showed that phenolic acids such as salicylic acid, p-hydroxybenzoic acid, cinnamic acid, vanilic acid and ferulic acid exhibited strong inhibitory effect on weeds^25^. Importantly, caffeic acid has also been reported to inhibit the development of *Phaseolus aureus* roots^26^. Therefore, caffeic acid may be one of the important components of *A. argyi*’s allelopathy.

### *A. argyi* revealed diverse inhibitory effects on different weeds

However, the water-soluble extract of *A. argyi* revealed diverse inhibitory effects on different weeds. The water extract significantly inhibited the *B. pekinensis* and *L.sativa* in the incubator experiment, whereas its effect on *O. sativa* was relatively weak (Table 2). Similarly, the order of susceptibility of each plant was *L. sativa* (Compositae) > *P. oleracea* (Portulaca oleracea) > *B. pekinensis* (Cruciferae) > *O. corniculata* (Oxalis) > *S. viridis* (Gramineae) > *O. sativa* (Gramineae) in pot experiments (Fig. 4). In field experiments, only one species of weed, *Eleusine indica* (Gramineae) could grow in the field treated with a high concentration of *A. argyi* powder (Fig. 5). Thus all these results indicated that different weeds have diverse sensitivities to allelopathic inhibition by *A. argyi* powder. Currently, commercial herbicides are usually divided into monocotyledon herbicides and dicotyledon herbicides. For example, Jinduer and Yanshu exhibited better inhibition effect on monocotyledon weeds than dicotyledon weeds^27^. 2, 4 -D butylate and starane particularly aim at dicotyledon weeds^28^. Based on our results, *A. argyi* could inhibit both dicotyledons and monocotyledons not only by seed germination but also by seedling growth. Therefore, *A. argyi* could be used as a raw material to develop herbicides to effectively control weed growth.

### Transcriptome revealed the possible allelopathic mechanisms

According to the current research, allelopathy can affect plant growth and development through multi-pathways, such as hormone levels^29^, the synthesis of polysaccharides (such as cellulose) in the cell wall^30^, photosynthesis^31^, respiration^32^, nucleic acid metabolism and protein synthesis^33^. For instance, the allelopathy of 1,4- and 1,8-cineole severely inhibited the various stages of mitosis, resulting in the cork-screw shaped morphological distortion, and inhibited the growth of roots and branches of two weedy plant species^30^. In addition, protocatechuic acid, the allelochemical in the aqueous extract solution of *Cerbera manghas L.*, could inhibit the reduction of quinone electrons and destroy the electron transfer in PSII , thus negatively affecting the photosynthesis of *Scrippsiella trochoidea*^31^. It provides an economic and eco-friendly method for the green control of harmful algal blooms.

In this study, we found that the pathways related to photosynthesis, nitrogen metabolism, porphyrin and chlorophyll metabolism are significantly inhibited after *A. argyi* treatment. These pathways are vital for plant growth and development. A series of key genes related to chlorophyll synthesis pathways including HEMA, HEML, CHLD, CHLH, CRD and CHLG were significantly reduced after treatment with *A.argyi* extract. In the Photosynthesis pathway, *A. argyi* extract could significantly down-regulate the expression of PsbY gene. The PsbY is a gene that has a novel manganese-binding, low-molecular-mass protein associated with PSII, this change might reduce the activity of PSII and the rate of electron transfer in *O.sativa*, resulting in a decrease in chlorophyll content. PetC is a key gene of cytochrome bf complex. Its main function is to participate in the cytochrome’s biological process in photosynthesis^34^. The PetC gene’s expression level was down-regulated under *A.argyi* extract treated compared with the control . Light harvesting complex II (LHCII) structure plays a crucial role in photosynthesis, the expression levels of Os04g38410 genes decreased means that normal photosynthesis is hindered (Fig. 8). The down-regulation of the genes in these pathway obstructs the basic processes of life activities such as carbon metabolism and nitrogen metabolism in plants. And we also observed that *A.argyi* treatment could decrease the content of chlorophyll and the biomass of the plants (Fig. 4), which is in consistence with our transcriptional analysis. In summary, the inhibition of *A. argyi*’s allelopathy on weeds might be owing to the suppression of the synthesis of chlorophyll and photosynthesis pathways.

### *A.argyi*’s allelopathy provides a valuable way of herbicide development

Analysis of raw materials with enhanced allelopathy inhibitory effects are valuable for the development of plant-derived herbicides. The allelopathy level of *A. argyi* is several times higher than allelopathic materials reported to date^35,36^, showing good herbicidal activity. Meanwhile, with the advantage of not producing adverse effects on field crops, *A.argyi* is worthy to be developed as a botanical herbicide, and suggesting it as the replacement for some chemical herbicides. The analysis of chemical composition and exploration of allelopathic mechanism about *A. argyi* have provided a more adequate and perfect theoretical basis for the ecological control of various weeds. However, pesticide preparation techniques and wider field application assessments are still needed to bring this herbicides into the reality. Importantly, we will create an ecological planting environment to make greater contribution to human health.

## Materials and methods

### Plant materials

The *A. argyi* was from Hubei Qichun. Then, the residue of the *A. argyi* leaf accounts for 90% percent of the total weight and is called as *A. argyi* leaf powder, after the production of moxa stick. The experimental seeds were *Brassica pekinensis*, *Lactuca sativa*, *Oryza sativa*, *Portulaca oleracea*, *Oxalis corniculata* and *Setaria viridis*.

### Chemical composition analysis of *A. argyi*

The chemical constituents of *A. argyi* were analyzed using UPLC-Q-TOF-MS method. 0.5 g of *A. argyi* leaves was mixed with 20 ml of ultra-pure water, 50% ethanol or pure ethanol independently and incubated overnight. After thorough oscillation, samples were subjected to ultrasonication for 1 h and centrifuged at 4000 rpm for 10 min. The supernatants were filtered through a 0.22 μm membrane and collected into a new tube. UPLC-Q-TOF-MS analysis was performed using an Waters Acquity I-Class ultra-performance liquid chromatography combined with a Xevo G2-S quadrupole time-of-flight mass spectrometer. Waters Acquity UPLC HSS T3 column (100 mm × 2.1 mm, 1.8 μm) was used. The specific chromatographic and mass spectrometric conditions were presented according to the method reported by Luo, D.D. et al^11^.

### Preparation of extracts of *A. argyi*

125 g of *A. argyi* leaf powder were soaked in 500 ml of water, 50% ethanol and pure ethanol, respectively, then treated with ultrasonic wave for 30 min every 12 h. 48 h later, the extracts were filtered with a double layer filter bag to obtain the crude extracts. The three types of crude extracts were centrifuged and the supernatant was collected. Then the ethanol extracts were treated with water bath to remove the ethanol and redissolved with ultra-pure water to a final concentration 0.25 g/ml. Finally, the three type extracts were diluted into 50 mg/ml, 100 mg/ml and 150 mg/ml with distilled deionized water, and stored in brown bottle, at 4 °C until use.

### Seeds germination assays

Seeds of *B. pekinensis*, *L. sativa* and *O. sativa* were sterilized with 0.2% sodium hypochlorite solution for 10 min and rinsed with distilled water for 3 times to avoid pathogen contamination. Twenty seeds from the above tested species were equidistantly placed in petri dish (diameter = 90 mm) with two layers of filter paper and then treated with 8 ml of the three extracts prepared from *A. argyi* at different concentrations (50 mg/ml, 100 mg/ml and 150 mg/ml) independently. Ultrapure water was used as the control, and 3 replicates of each treatment group were established. The dishes were then cultured in universal environmental test chamber at a constant temperature of 25 ± 0.5 °C, 85% humidity, and a controlled 12 h light/12 h dark cycle. Two milliliters of corresponding extract were added every 48 h to maintain the humidity of the filter paper in the petri dish. The number of the germinated seeds was counted from the second day after treatment and the count lasted for one week. In addition, the root length, stem length and biomass of each treatment were also measured.

### Pot experiment

For soil preparation, *A. argyi* powder was mixed into sand soil at the ratio of 100:0, 100:2, 100:4, and 100:8, separately. 800 g soil poured into plastic bowls with upper mouth diameter of 14.5 cm, lower mouth diameter of 10.5 cm and height of 9 cm. Then equal amount of water was added into each treatment group to soak the soil and seeds were sown 2 days later. Fifty seeds of *B. pekinensis*, *L. sativa*, *O. sativa*, *P. oleracea*, *O. corniculata* and *S. viridis* were sown independently in each bowl with three biological repeats. The germination rates and plant heights of *B. pekinensis*, *L. sativa* and *O. sativa* were measured at 9 days post treatment, and same data were recorded in *P. oleracea*, *O. corniculata*, and *S. viridis* at 13 days post treatment.

### Field trial

The medicinal botanical garden of Hubei University of Chinese Medicine was selected as the experimental site to observe the effect of *A. argyi* on weeds growth. The experimental land in which *Chrysanthemum morifolium* seedlings were transplanted after spring tillage was divided into an average of 12 plots. Each plot was 5 m^2^, with shallow trenches separating each plots. *A. argyi* powder was evenly applied to the plots at the concentrations of 0 kg/m^2^, 0.1 kg/m^2^ and 0.2 kg/m^2^ with four biological repeats. One month later, the effects of *A. argyi* powder on weed varieties, quantity and biomass in each treatment groups were investigated. In addition, the growth and yield of *C. morifolium* were also determined.

### RNA isolation and RNA-seq

*O.sativa* was selected as the test plant, and the seeds were placed in filter paper roll in Hoagland nutrient solution containing 0.0 g/ml (LCK), 0.03 g/ml (LT) of *A. argyi* powder water-cooled extract, respectively, and sampled for use 7 days later with three independent biological repeats. Total RNA was extracted from samples by using CTAB-PBIOZOL methods according to the manual instructions^37^. RNA libraries were generated and sequenced on BGISEQ500 platform in Beijing Genomics Institute (BGI) in Shenzhen.

### Sequencing analysis and differential expression analysis

The sequencing data was filtered with SOAPnuke (v1.5.2)^38^ by (1) Removing reads containing sequencing adapter; (2) Removing reads whose low-quality base ratio (base quality less than or equal to 5) is more than 20%; (3) Removing reads whose unknown base ('N' base) ratio is more than 5%, afterwards clean reads were obtained and stored in FASTQ format. The clean reads were mapped to the reference genome using HISAT2 (v2.0.4)^39^. Bowtie2 (v2.2.5)^40^ was applied to align the clean reads to the reference coding gene set, then expression level of gene was calculated by RSEM (v1.2.12)^41^. The heatmap was drawn by pheatmap (v1.0.8) according to the gene expression in different samples. Essentially, differential expression analysis was performed using the DESeq2(v1.4.5)^42^. To take insight to the change of phenotype, KEGG (https://www.kegg.jp/) enrichment analysis of annotated different expressed gene was performed by Phyper (https://en.wikipedia.org/wiki/Hypergeometric_distribution) based on Hypergeometric test.

### Real-time quantitative PCR (RT-qPCR)

*O.sativa* was selected as the test plant, and the seeds were placed in afilter paper roll in Hoagland nutrient solution containing 0.0 g/ml(CK), 0.01 g/ml(L), 0.03 g/ml(M), 0.05 g/ml(H) of *A. argyi* powder extract, respectively. After 21 days of culture, the expression patterns of 9 genes associated with photosynthesis related to *O.sativa* were analyzed using RT-qPCR (gene-specific primers are provided in supplement Table 1). Total RNA was isolated from the leaves using TRIzol (Tiangen Bio Co.,Ltd., Beijing, China), and converted to cDNA using MLV reverse transcriptase (Promega Corporation, USA). RT-qPCR was conducted using RealUniversal Color PreMix(SYBR Green) according to the manufacturer’s instructions (Tiangen, Beijing, China). The actin gene (5′-TGCTATGTACGTCGCCATCCAG-3′ and 5′-AATGAGTAACCACGCTCCGTCA -3′) was used as an internal standard. The PCR program quoted Zhang, Q. et al^15^ and modified it as follows: 15 min of pre-incubation at 95 °C, followed by 40 cycles of 10 s at 95 °C and 35 s at 60 °C and steps for melting curve generation (5 s at 95 °C, 60 s at 65 °C, and finally at 97 °C). The relative gene expression of each target gene was determined using the 2^−ΔΔt^ method.

#### Statistical analysis

Mass spectrometry data were recorded and analyzed using Masslynx 4.1 software. Mass spectrometry data were obtained using Progenesis QI V2.0 (Waters Corp., Milford, USA) for processing.

The germination rate (GR), germination speed index (GSI) and germination index (GI) were used to determine the germination according to the methods previously^43^. GR = (number of germinated seeds/total number of tested seeds) × 100%. GSI = (7X_1_ + 6X_2_ + 5X_3_ + 4X_4_ + 3X_5_ + 2X_6_ + X_7_) (where X is the total number of germinated seeds after X days). GI = Σ (Gt/Dt), where Gt represents the germination numbers on day t and Dt represents the corresponding germination days.

The allelopathy response index (RI) was calculated to quantify the type and intensity of allelopathy as the method described previously^44^ using the following formulas: when T ≥ C, RI = 1-C/T; when T < C, RI = T/C-1 (T < C). C is the control value and T is the treatment value, RI > 0 represents a stimulatory effect, RI < 0 represents an inhibitory effect, and the absolute value is consistent with the allelopathy intensity. Synthetic allelopathy (SE) was evaluated by calculating the arithmetic mean value of RI for 6 parameters, including the germination rate, germination speed index, germination index, root length, stem length and biomass.

The experimental data recorded from the study were analyzed using GraphPad Prism 5 and SPSS24.0 software. T-test and One-way Analysis of Variance (ANOVA) were used to analyze the significant difference between different treatment groups.

## Supplementary Information

Supplementary Information.
