# Supplementary material for: Allelopathic effect of Artemisia argyi on the germination and growth of various weeds
Source: Sci Rep. 2021 Feb 22;11:4303. doi: 10.1038/s41598-021-83752-6 (PMC7900198; doi:10.1038/s41598-021-83752-6)
Supplement: Supplementary file 1 — Supplementary Information. [file 41598_2021_83752_MOESM1_ESM.pdf]

Allelopathic Effect of *Artemisia argyi* on the Germination and Growth of Various Weeds

Jinxin Li[1]&Le Chen[1], Qiaohuan Chen[1], Yuhuan Miao[1], Zheng Peng[1], Bisheng Huang[1], Lanping Guo[2]，Dahui Liu[1*]&Hongzhi Du[1*]

Jinxin Li and Le Chen are the co-first authors for this manuscript

1 Hubei Provincial Key Laboratory of Traditional Chinese Medicine Resources and Traditional Chinese Medicine Chemistry, Hubei University of Chinese Medicine, Wuhan, China

2 National Resource Center for Chinese Materia Medica, China Academy of Chinese Medical Science, Beijing, China

* Correspondence: Dahui Liu; E-mail: liudahui@hbtcm.edu.cn

Hongzhi Du；E-mail: dhz3163@hbtcm.edu.cn

Table 1 The sequence of gene-specific primers for PCR used for detecting gene expression in the leaves of *O.sativa*.

Gene name	F（5'-3')	R（5'-3')
HEMA	GATGCAATCACTGCTGGAAAGCGT	CCATCTTGCCAGCACCAATCAACA
HEML	AGAACAAAGGGCAGATTGCTGCTG	TGTTTCGTCAAGTCACGGAGAGCA
CHLD	TAGCACAGCTGTCAGAGTGGGTTT	TTGCCAGCCACCTCAAGTATCTCA
CHLH	GCACGGGAACTTGGCGTTTCATTA	ACATGTCCTGGAGCTGCTTCTCAT
CRD	TGGATCTAACATGACACGCACCCA	ACTGTAACGGCATTCTTCTCCGGT
CHLG	CCAGCCACTGATGAAAGCAGCAAT	AGAGCGCTAATACACTCGCGAACA
CAO	ACACCTTCATCTGGGCTTCAAGGA	AGATGCGTCGAACATTGCTTGGTG
PsbY	CGCAGCGACATTGCACTCACC	TGACGCCTTCTTCTTCCTCCTCTT
PetC	GCACCACTTCGTCCTCAACCA	TGTCAGGCAGCAAGTTCTTCACC
Os04g38410	GTCTACATCCCGGACACCGACAA	GCTACTGCTCCTCCGACCTCCAAT

Table 2 The abbreviations and units for individual parameters of manuscript

Full name	abbreviations	individual parameters	units
Artemisia argyi	*A. argyi*	root length	cm
Brassica pekinensis	*B. pekinensis*	stem length	cm
Lactuca sativa	*L. sativa*	biomass	g/dish
Oryza sativa	*O. sativa*	germination rate	%
Portulaca oleracea	*P. oleracea*	plant's height	cm
Oxalis corniculata	*O. corniculata*		
Setaria viridis	*S. viridis*		
Chrysanthemum morifolium	*C. morifolium*		
RealTime QuantitativePCR	RT-qPCR		
Ultra-performance liquid chromatography coupled to quadrupole time-of-flight mass spectrometry	UPLC-Q-TOF-MS		
differentially expressed genes	DEGs		
Kyoto Encyclopedia of Genes and Genomes	KEGG		
germination rate	GR		
germination speed index	GSI		
germination index	GI		
response index	RI		
synthetic allelopathy	SE		

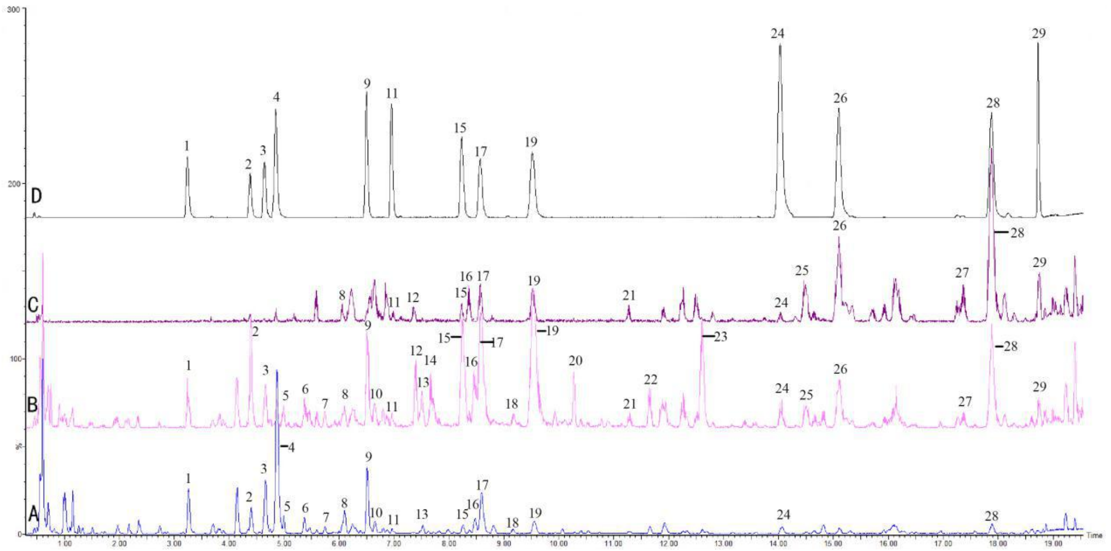

Figure 1 The fingerprint of different solvent extracts of *A. argyi* in UPLC-Q-TOF-MS. (A)the water-soluble extract, （B） the 50% ethanol extract, (C) the pure ethanol extract, (D) the standard substance. The mass spectrometry data such as total ion chromatograms, molecular ion peaks and secondary fragment ions were collected by Masslynx 4.1 software and processed with ProGenesis QI V2.0 (Waters Corp., Milford, USA).
